# Systematic review on the use of artificial intelligence to identify anatomical structures during laparoscopic cholecystectomy: a tool towards the future

**DOI:** 10.1007/s00423-025-03651-6

**Published:** 2025-03-18

**Authors:** Diletta Corallino, Andrea Balla, Diego Coletta, Daniela Pacella, Mauro Podda, Annamaria Pronio, Monica Ortenzi, Francesca Ratti, Salvador Morales-Conde, Pierpaolo Sileri, Luca Aldrighetti

**Affiliations:** 1 Hepatobiliary Surgery Division, IRCCS San Raffaele Scientific Institute, Via Olgettina 60, 20132 Milan, Italy; 2 Department of General Surgery and Surgical Specialties “Paride Stefanini”, Sapienza University of Rome, Viale del Policlinico 155, 00161 Rome, Italy; 3 Department of General and Digestive Surgery, University Hospital Virgen Macarena, University of Sevilla, Seville, Spain; 4 Unit of General and Digestive Surgery, Hospital Quirónsalud Sagrado Corazón, Seville, Spain; 5 General and Hepatopancreatobiliary Surgery, IRCCS Regina Elena National Cancer Institute, Rome, Italy; 6 Department of Public Health, University of Naples Federico II, Naples, Italy; 7 Department of Surgical Science, University of Cagliari, Cagliari, Italy; 8 Department of General Surgery and Surgical Specialties, Sapienza University of Rome, Viale del Policlinico 155, 00161 Rome, Italy; 9 Department of General and Emergency Surgery, Polytechnic University of Marche, Ancona, Italy; 10 Hepatobiliary Surgery Division, IRCCS San Raffaele Scientific Institute, Faculty of Medicine and Surgery, Vita-Salute San Raffaele University, Via Olgettina 60, 20132 Milan, Italy; 11 Coloproctology and Inflammatory Bowel Disease Surgery Unit, IRCCS San Raffaele Scientific Institute, Faculty of Medicine and Surgery, Vita-Salute University, Via Olgettina 60, 20132 Milan, Italy

**Keywords:** Artificial intelligence, Laparoscopic cholecystectomy, Minimally invasive surgery, Bile duct injuries

## Abstract

**Purpose:**

Bile duct injury (BDI) during laparoscopic cholecystectomy (LC) is a dreaded complication. Artificial intelligence (AI) has recently been introduced in surgery. This systematic review aims to investigate whether AI can guide surgeons in identifying anatomical structures to facilitate safer dissection during LC.

**Methods:**

Following PROSPERO registration CRD-42023478754, a Preferred Reporting Items for Systematic Reviews and Meta-Analyses (PRISMA)-compliant systematic search of MEDLINE (via PubMed), EMBASE, and Web of Science databases was conducted**.**

**Results:**

Out of 2304 articles identified, twenty-five were included in the analysis. The mean average precision for biliary structures detection reported in the included studies reaches 98%. The mean intersection over union ranges from 0.5 to 0.7, and the mean Dice/F1 spatial correlation index was greater than 0.7/1. AI system provided a change in the annotations in 27% of the cases, and 70% of these shifts were considered safer changes. The contribution to preventing BDI was reported at 3.65/4.

**Conclusions:**

Although studies on the use of AI during LC are few and very heterogeneous, AI has the potential to identify anatomical structures, thereby guiding surgeons towards safer LC procedures.

**Supplementary Information:**

The online version contains supplementary material available at 10.1007/s00423-025-03651-6.

## Introduction

Laparoscopic cholecystectomy (LC) is the gold standard treatment for symptomatic cholecystolithiasis and one of the most performed surgical procedures worldwide, both in elective and emergency settings and by senior and trainee surgeons [1–4]. The most dreaded complication of LC is bile duct injuries (BDIs) and a primary cause of BDI is a misunderstanding of the anatomical structures [1, 5]. The iatrogenic BDI rate after LC is higher than open cholecystectomy and ranges between 0.4 and 1.5% [5]. BDIs are responsible for further surgical, endoscopic, or radiological procedures, increasing cost management of patients [5, 6].

Several optimal strategies for prevention of the BDIs are reported in the literature, which include the systematic use of the Critical View of Safety (CVS), the bailout procedures, such as sob-total cholecystectomy and many others [7]. The use of indocyanine green (ICG) fluorescence and the possibility to perform a fluorescent cholangiography (FC) during LC allows the visualization of extrahepatic biliary structures and facilitates intraoperatively dissection, aiming to reduce the BDI risk [8–19]. However, in the case of obese patients or acute cholecystitis, misinterpretation of the biliary anatomy can still occur, with a high risk of BDI [20–23].

Artificial intelligence (AI) has been recently introduced in MIS [24–28]. Computer vision is a field of AI that focuses on identifying and accurately analysing digital images, videos and other input, simulating and optimizing human recognition ability [25–28]. Although the use of deep learning and computer vision in diagnostics, especially endoscopic, is now well established, its real-time application during surgery is still in the earliest stages [24–28].

The present systematic review aims to investigate whether AI can guide surgeons in identifying anatomical structures to facilitate safer dissections during LC.

## Materials and methods

Institutional review board approval and informed consent from participants were unnecessary for the present study. This study was registered in the International Prospective Register of Systematic Reviews (PROSPERO; CRD-42023478754).

### Search strategy

A systematic review of published articles according to the Preferred Reporting Items for Systematic Review and Meta-Analysis (PRISMA) Statement 2020 [29] and according to the Cochrane Handbook for systematic reviews of interventions was conducted [30]. The aim of the present study was to explore the potential of AI to improve the safety of LC by aiding surgeons in the identification of anatomical structures.

The PICO question was generated from a discussion within the authors (D.C., D.C., D.P., A.B.).

The following PICO question was adopted:

- P(opulation). Patients undergoing LC.
- I(ntervention). Identification of the anatomical landmarks during LC by AI.
- C(omparison). Identification of the anatomical landmarks during LC without AI.
- O(utcomes). Feasibility of anatomical structures identification during LC using AI.

### Study identification

A computerized search was performed in MEDLINE (via PubMed), EMBASE and Web of Science databases for articles published up to 30/09/2024 [31], without language restrictions (Supplementary Material-Table 1). Reviews, systematic reviews, meta-analysis, comments, case reports, congress abstracts, correspondence and letters to editor, editorials, technical surgical notes, imaging studies and studies with animals’ involvement were excluded. For a more objective evaluation, studies in which AI was used to identify adverse effects, timing, operators, and surgical tools, were excluded from this systematic review.

### Eligibility criteria, screening process, and data extraction

All articles in which AI was used to anatomical structures identification, groups of anatomical structures that configure safe/unsafe dissection zones, or CVS assessment during LC were eligible for inclusion in the present study. Two independent reviewers (D.C. and D.C.) conducted the screening process and data extraction in a double-masked fashion. Discrepancies were resolved with a discussion with a third reviewer (A.B.). Data collected from each study included the following predefined items: (1) Study identifier (first author, year of publication); (2) Study design; (3) Population; (4) Type of dataset; (5) Inclusion and exclusion criteria; (6) Anatomical structures identified; (7) AI model; (8) “Ground truth” establishment; (9) Validation and application of the model; (10) Outcome measure; (11) Main results; (12) Conclusion; (13) Risk of bias/quality assessment. Data were stored in the Microsoft Excel program (Microsoft Corporation, Redmond, Washington, USA).

### Outcomes of interest

According to the PICO criteria, the aim of this systematic review is to report the current evidence of the use of AI for anatomical structures identification during LC.

Structure identification concerns the hepatocystic anatomy: gallbladder, cystic duct (CD), cystic artery (CA), cystic plate (CP), common bile duct (CBD), hepatocystic triangle (HCT), lower edge of the left medial liver segment (LEoLMLS), Rouviere’s sulcus (RS) [27, 32–55]. Some authors distinguish the anatomical identification into safe and dangerous zone for dissection, called “Go” and “No-Go” zone, respectively [36, 37, 45, 54]. The “Go” zone was defined as the area located within the HCT (closer to the inferior edge of the gallbladder) that is deemed safe to proceed with dissection with a low probability of causing a major BDI. The “No-Go” zone was defined as the deeper region within the HCT, where further dissection was deemed unnecessary and dangerous with an unacceptable probability of causing a major BDI. The “No-Go” zone also includes the hepatoduodenal ligament, liver hilum, and all structures inferiorly [27, 35]. Non-segmented regions of dissection are deemed as “in-between” (i.e., neither Go, or No-Go) zone [45].

As CVS is based on the proper identification of anatomical structures, studies involving CVS assessment were included in this systematic review [33, 27, 35, 36, 39, 41, 44, 45, 47, 48, 50–52]. The achievement of CVS is based on three criteria: the 2-structure criterion (CA and CD entering the gallbladder), the HCT criterion (a carefully dissected HCT), and the CP criterion (the division of the lower third of the gallbladder from the CP) [27].

Anatomical identification and CVS assessment by AI could be achieved in several ways [32–34, 27, 35–54]. Semantic segmentation [36, 37, 45, 54], computer vision model for workflow analysis [27, 35, 36], Deep Convolutional Neural Network (DCNN) and Graph neural networks (GNNs) [33, 41, 44, 45], bounding boxes [32, 34, 39, 41, 42, 48] and MIL models [38, 51] are some examples.

The AI model performance was compared to surgical annotations [32, 27, 33–55]. It was evaluated in terms of mean average precision (mAP), mean or balanced accuracy (b-ACC), Area Under the Curve (AUC), sensitivity or recall, specificity, negative predictive value (NPV), positive predictive value (PPV), detection rate (DR) and positive ratios with PMLA (partial matches between landmark detection and annotation) [32–34, 27, 35–55]. mAP evaluates the overall performance of the model, defined as the average precision across all recall values between 0 and 1, while bACC is the average of sensitivity and specificity for the best model selected using the validation set [36]. AUC is an effective metric to summarize the overall accuracy of the test. It takes values from 0 to 1, where a value of 0 indicates a perfectly inaccurate test and a value of 1 reflects a perfectly accurate test [56]. In general, an AUC from 0.7 to 0.8 is considered acceptable, 0.8 to 0.9 is considered excellent, and more than 0.9 is considered outstanding [51]. The performance of the segmentation network was measured with mean intersection over union (IOU) and Dice/F1 score, which are two commonly used metrics in computer vision to quantify the percentage of overlap between the segmentation output and the “ground truth” [37]. The IOU value will be closer to 1 as much as the segmentation is correct (and therefore the greater the overlap area), while the F1 score is calculated as the harmonic mean of the precision and recall scores. It ranges from 0–100%, and a higher F1 score denotes a better-quality classifier. [35, 36, 40, 43]. In case of CVS achievement, the validity of the AI annotations of CVS component (AI-surgeon inter-rater agreement) was assessed with Cohen’s kappa, percentage of agreement and Gwet’s AC2, a weighted measure that adjusts for scales with high probability of random agreement and represented by a collapsed dichotomous scoring rubric of “poor—unsafe” vs. “adequate/excellent—safe”. Gwet’s AC2 metric ranges from 0 to 100%: excellent (81% to 100%), very good (61% to 80%), moderate (41% to 60%), and poor (< 40%). [33, 27, 47, 50, 57].

### Risk of bias assessment in the included studies

Risk of bias of the included studies was assessed three authors (D.C., D.C., A.B.) using the revised Cochrane risk-of-bias tool for randomized trials (RoB 2) and the risk of bias in non-randomized studies for interventions (ROBINS-I) tool [58, 59]

### Grading the quality of evidence

According to the Grading of Recommendations, Assessment, Development, and Evaluations (GRADE) approach, two authors (D.C., A.B.) independently evaluated the quality of evidence for imprecision, inconsistency, indirectness, and publication bias [60]. Moreover, the quality of evidence was classified as very low, low, moderate, or high [60]. Subsequently, a summary table was created using the GRADE profiler software (version 3.6.1) (available at: http://www.gradeworkinggroup.org/) [60].

## Results

**Fig. 1:**
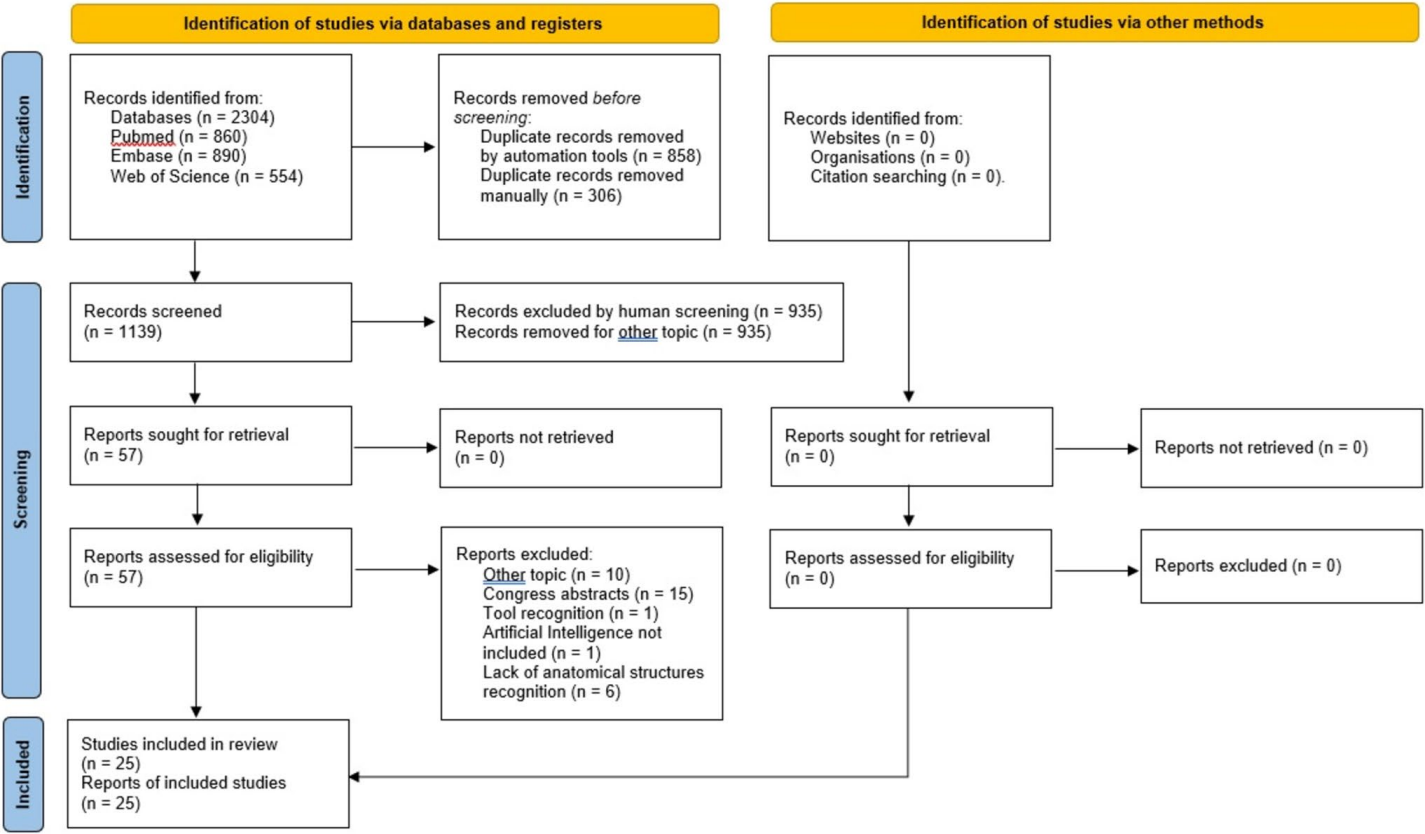
Preferred Reporting Items for Systematic Review and Meta-Analysis (PRISMA) flow diagram [29]

The search revealed 2304 articles, of these, 1164 were eliminated because they were duplicates. Of the remaining 1139 articles, 935 were excluded after screening title and abstract because they did not meet the inclusion criteria. Fifty-seven articles were thoroughly analysed, and 33 further articles were excluded. Finally, twenty-five articles published between June 2020 and September 2024 were included [27, 32–43], as shown in the PRISMA flow diagram (Fig. 1) [29].

Table 1 reports studies characteristics and results, while the risk of bias based on ROBIN-I and RoB 2 of the included articles is reported in Tables 2 and 3. The assessment of evidence according to the GRADE method of the included articles is reported in Supplementary Material-Table 2.

**Table 1:** Summary of the 25 included studies

Author (year), [reference]	Aim	Dataset (n of videos)	Exclusion criteria	Anatomical structures identified	AI model	Ground truth establishment/annotations	Outcome
Tokuyasu et al*.* [32]	identification of individual anatomical structures	99	high inflammation and fibrosis, bleeding, or less‑visible landmarks	CD, CBD, LEoLMLS, RS	YOLOv3	two expert surgeons (> 200 LC)	mAP for CBD:32%; CD: 7%, LEoLMLS: 3%; RS: 10%
Korndorffer et al*.* [33]	CVS assessment	385	None	HCT, CP, CD, CD + CA	ConvNet; 3D DCNN	Eight boardcertified surgeons	% of agreement: HCT 81%, cystic plate 84%, CD + CA 81%, CVS 93% Kappa: HCT 0.6, cystic plate 0.6, CD + CA 0.4, CVS 0.6
Inomata et al*.* [34]	identification of individual anatomical structures	99	high inflammation and fibrosis, bleeding, or less‑visible landmarks	CD, CBD, LEoLMLS, RS	YOLOv3	two expert surgeons (> 200 LC)	High DR of landmarks in case of mild to moderate inflammation
Mascagni et al*.* [27]	CVS assessment	155	Incomplete recordings, bailout procedures, IOC	HCT, CP, CD, CD + CA	EndoDigest	two independent HPB surgeons	Average error for cystic duct division identification:1.9%, CVS assessment: 91% Inter-rater agreement for CVS 85%, AC + CD 91%, HCT 82%, CP 84%
Mascagni et al*.* [35]	CVS assessment	144	Incomplete recordings, bailout procedures, IOC	HCT, CP, CD, CD + CA	EndoDigest	two independent HPB surgeons	75% of agreement for CVS achievement
Mascagni et al*.* [36]	CVS assessment	201	abnormal hepatocystic anatomy and absence of anterior view of CVS	Gallbladder, CD, CA, CP, HCT	DeepCVS	one surgeon in training and one HPB surgeon, in case of disagreement a senior HPB surgeon mediated	Inter-rater agreement for CVS 82%; CA + CD 93%; HCT 68%; CP 74% CVS mAP: 72%; CVS bACC: 71%; Se: 70%; Sp: 72%; Mean IOU: 0.7
Madani et al*.* [37]	identification of individual anatomical structures and safe/dangerous zones of dissection	308	subtotal and top-down approach LC	Go zone, No-Go zone and target anatomy (gallbladder, liver, HCT)	ResNet50 (GoNoGoNet CholeNet)	three acute care and MIS surgeon and one high-volume HPB surgeon	Go zone: ACC 94%; Se 69%; Sp 94%; NPV 96%; PPV 74%; IOU 0.5; F1 score 70% No-Go zone: ACC 95%; Se 80%; Sp 98%; NPV 97%; PPV 86%; IOU 0.7; F1 score 83% HCT: ACC 93%; Se 80%; Sp 95%; NPV 96%; PPV 78%; IOU 0.7; F1 score 79%
Laplante et al*.* [38]	identification of safe/dangerous zones of dissection in comparison to expert surgeons	25	top-down approach LC, and poor visualization of the hepatocystic triangle	Go zone, No-Go zone	GoNoGoNet	six high-volume expert surgeons from the SAGES Safe Cholecystectomy Task Force	Go zone: ACC 92%; Se 52%; Sp 97%; NPV70%; PPV 94%; IOU > 0.4; F1 score 58% No-Go zone: ACC 92%; Se 80%; Sp 95%; NPV 95%; PPV 84%; IOU > 0.7; F1 score 80%
Colbeci et al*.* [39]	CVS assessment	556	None	HCT, CP, CD, CD + CA	MIL model	A group of surgeons	ACC: HCT 83%, cystic plate 83%, CA + CD 82%, mean ACC 83% CVS: ACC 84%, Sp 87%, Se 72%, F1 score 66%
Nakanuma et al. [40]	identification of individual anatomical structures	10	high inflammation and fibrosis, bleeding, or less‑visible landmarks	CD, CBD, LEoLMLS, RS	YOLOv3	three expert surgeons (not belonging to the research group)	F1 score for CBD 72%; CD 49%; S4 46%; RS 66%; bACC 4.2/5
Ban et al*.* (2023) [41]	CVS assessment	100	None	HCT, CP, CD, liver, CD + CA	ConceptNet-Resnet50; ConceptNet-VIT	clinical CVS experts	bACC: CVS 67%, CA 55%, CD 55%, liver 60%, CP 56%, CA + CD 54%
Fujinaga et al*.* [42]	identification of individual anatomical structures and prevention of BDIs	30	conversion to open surgery	CD, CBD, LEoLMLS, RS	Cross-AI (YOLOv3 + EfficientNet-B5 and a SAM optimizer)	two expert surgeons; IEC: three surgeons involved in the study; EEC three certified surgeons from multiple institutions	F1score for CBD 45%, CD 20%, S4 27%, RS 30%; PMLA for CBD 84%, CD 77%, S4 87%, RS 70%; ACC for CBD and CD 3.8/4 and 3.7/4, respectively. The contribution to preventing BDI was at 3.7/4
Endo et al. [43]	evaluation of the influence of the AI system on the identification of individual anatomical structures	95	severe inflammation and abnormal biliary anatomy	RS, LEoLMLS, CD and EHBD	YOLOv3	four beginners and four expert surgeons	27% of changed annotations (70% safer changes)
Kawamura et al. [44]	CVS assessment	72	severe cholecystitis	CD, CA, CP, HT	EfficientNet-B5 and a SAM optimizer	a board-certified surgeon in gastroenterology A JSES board certified surgeon performed the final confirmation of the annotation data	mAP:97%; Se:74%; F1 score:83%; Sp:97%; ACC:83%
Alkhamaiseh et al. [45]	CVS assessment	200	poor image quality (bleeding, less visible landmarks, anatomical differences) and no- standard LC	HCT, CP, CD, CD + CA	Auto-Encoder; U-Net	two expert surgeons and three 5th-year resident	ACC 92%; Mean IOU: 0.75
Khalid et al. [46]	identification of safe/dangerous zones of dissection in LC with BDIs	22	poor visual quality	Go zone, No-Go zone, or neither (i.e., in between the two)	U-Net	two independent high-volume surgeons (members of the SAGES Safe Cholecystectomy Task Force)	LC videos with BDIs had a significantly greater proportion of dissection occurring outside of the Go zone compared to LC videos in the Control group (*p* < 0.01)
Adrales et al. [47]	CVS assessment	36	None	HCT, CP, CA, CD	LC-CVS OPSA	12 expert LC surgeon	Inter-rater reliability: Gwet’s AC2: HCT 41%; CP 50%; CA 54%; CD 72%. Total agreement: HCT 76%; CP 78%; CA 80%; CD 86%
Hedge et al. [48]	identification of individual anatomical structures and CVS assessment	160	None	HCT, CP, CD, CD + CA	3D-CNN	three expert surgeons	CVS: precision 83%; Se 85%; F1 score:84% CD + CA: precision 77%; Se 65%; F1 score:71%
Smithmaitrie et al. [49]	identification of individual anatomical structures and visualization of guided dissection line	40	None	LEoLMLS, RS	YOLOv7	three independent surgeons	LEoLMLS + RS: pr 92%; Se:87%, mAP 91% IOU 0.5. Mean surgeons’ acceptances of the guided line dissection: 96%
Petracchi et al. [50]	CVS assessment	40	Non-elective LC performed for reasons different from symptomatic gallstones and Tokyo 1 cholecystitis	CD, CA and CVS	PyCharm, Google Colab Pro and YOLOv8	three blinded surgeons	100% accordance for CVS achievement
Leifman et al. [51]	CVS assessment	40	None	CVS: HCT, CP, CD, CA, liver	SpineNet-49, Resnet50	three independent surgeons	Sp 100%; Se 97%; CVS AUC 0.9 (CD and CA: 0.89, HCT: 0.96, CP: 0.88, liver: 0.9)
Fried et al. [52]	CVS assessment	279	None	CVS: HCT, CP, CD, CA	MIL model	two expert surgeons	ACC 95%; CA and CD visibility rise from 42 to 75% (*p* < 0.001) thanks to AI-CVS achievement
Tashiro et al. [53]	LCT with AI and anatomical structures with ICG	1	LC without ICG	LCT, CD, gallbladder, liver	Eureka	-	LCT identification: IOU 0.6; F1 score:60%. The combination of AI and ICG may reduce BDI rate
Wu et al. [54]	CVS assessment and improving in surgical performance	90	Other concomitant procedures, patients with severe comorbidities, suspect of gallbladder malignancy and Parkland score ≥ 3	HCT, CP, CD, CD + CA	SmartCoach	hepatobiliary experts	Significant improvement in CVS achievement in AI-enhanced feedback group as compared to self-learning group (11% to 78%, *p* = 0.02
Protserov et al. (2024) [55]	identification of safe/dangerous zones of dissection	314	subtotal and top-down approach LC	Go zone, No-Go zone	U-Net and SegFormer	A panel of expert high-volume surgeons for Go and No-Go zone, separately	U-Net: Go-zone: F1 score 57%; precision 48%; Se 82%; RAE + 92% No-Go-zone: F1 score 76%; precision 68%; Se 92%; RAE + 47% SegFormer: Go-zone: F1 score 60%; precision 53%; Se 75%; RAE + 48% No-Go-zone: F1 score 76%; precision 68%; Se 92%; RAE + 46%

*AI* Artificial intelligence; *BDI* Bile duct injury; *CD* Cystic duct; *CA* Cystic artery; *CBD* Common bile duct; *LEoLMLS* Lower edge of the left medial liver segment; *RS* Rouviere’s sulcus; *HCT* hepatocystic triangle; *YOLOv3* You only look once version 3; *LC* Laparoscopic cholecystectomy; *mAP* Mean average precision; *DR* Detection rate; *CVS* Critical view of Strasberg; *CP* Cystic plate; *HPB* Hepato-pancreato-biliary; *DCNN* Deep Convolutional Neural Network; *IOU* Intersection over union; *bACC* Balanced accuracy; *ACC* Accuracy; *Se* Sensitivity; *Sp* Specificity; *MIS* Minimally invasive surgery; *NPV* Negative predictive value; *PPV* Positive predictive value; *IOC* IntraOperative Cholangiography; *SAGES* Society of American Gastrointestinal and Endoscopic Surgeons; *MIL* Multi Instance Learning; *EEC* External evaluation committee; *EHBD* Extrahepatic bile duct; *S4* Segment 4; *SAM* Sharpness-Aware Minimization; *IEC* Internal evaluation committee; *JSES* Japanese Society for Endoscopic Surgery; *PMLA* Partial matches between landmark detection and annotation; *AUC* Area Under the Curve; *CNN* Convolutional Neural Network; *LCT* Loose connective tissue; *ICG* Indocyanine green; *RAE* Relative-Area-Error

**Table 2:** Assessment of risk of bias of the included articles based on Risk of Bias in Non-randomised Studies – of Interventions (ROBINS-I) [58]

Author, year, type of study	Bias due to confounding	Bias in selection participants	Bias in classification of interventions	Bias due to deviations from intended interventions	Bias due to missing data	Bias in measurement of outcomes	Bias in selection of reported result	Overall
Tokuyasu et al*.* 2020, ambispective [32]	Moderate	Moderate	Low	Moderate	Moderate	Moderate	Moderate	Moderate
Korndorffer et al. 2020, retrospective [33]	Low	Low	Low	Low	Low	Moderate	Low	Low
Inomata et al*.* 2021, ambispective [34]	Low	Moderate	Low	Low	Moderate	Moderate	Moderate	Moderate
Mascagni et al*.* 2021, retrospective [27]	Low	Moderate	Low	Low	Low	Moderate	Low	Low
Mascagni et al*.* 2022, retrospective [35]	Low	Moderate	Low	Low	Low	Moderate	Low	Low
Mascagni et al*.* 2022, retrospective [36]	Low	Moderate	Moderate	Moderate	Low	Low	Low	Low
Madani et al*.* 2022, retrospective [37]	Low	Low	Low	Moderate	Low	Moderate	Moderate	Low
Laplante et al*.* 2022, prospective [38]	Low	Low	Low	Moderate	Low	Moderate	Moderate	Low
Colbeci et al*.* 2022, retrospective [39]	Low	Low	Low	Low	Low	Moderate	Low	Low
Nakanuma et al*.* 2022, prospective [40]	Low	Low	Low	Low	Low	Moderate	Low	Low
Ban et al*.* 2023, retrospective [41]	Low	Moderate	Moderate	Moderate	Low	Low	Low	Moderate
Endo et al*.* 2022, retrospective [42]	Low	Low	Low	Low	Low	Moderate	Low	Low
Fujinaga et al*.* 2022, prospective [43]	Low	Low	Low	Low	Low	Moderate	Low	Low
Kawamura et al*.* 2022, prospective [44]	Low	Low	Low	Low	Low	Moderate	Low	Low
Alkhamaiseh et al*.* 2023, retrospective [45]	Low	Low	Low	Low	Low	Moderate	Low	Low
Khalid et al*.* 2022, retrospective [46]	Low	Low	Low	Low	Low	Moderate	Low	Low
Adrales et al*.* 2023, retrospective [47]	Low	Moderate	Low	Low	Low	Moderate	Low	Low
Hedge et al. 2023, retrospective [48]	Low	Low	Low	Low	Low	Moderate	Low	Low
Smithmaitrie et al. 2024, ambispective [49]	Low	Moderate	Low	Low	Low	Moderate	Low	Low
Petracchi et al. 2024, prospective [50]	Low	Low	Low	Low	Low	Moderate	Low	Low
Leifman et al. 2024, prospective [51]	Low	Moderate	Low	Low	Low	Moderate	Low	Low
Fried et al*.* 2024, ambispective [52]	Low	Moderate	Low	Low	Low	Moderate	Low	Low
Tashiro et al. 2024, ambispective [53]	Moderate	Moderate	Moderate	Moderate	Moderate	Moderate	Moderate	Moderate
Protserov et al. 2024, ambispective [55]	Low	Moderate	Low	Low	Low	Moderate	Low	Low

Low: low risk of bias (the study is comparable to a randomised trial). Moderate: moderate risk of bias (the study provides sound evidence for a nonrandomised study but cannot be considered comparable to a randomised trial). Serious: serious risk of bias (the study has important problems)

**Table 3:**
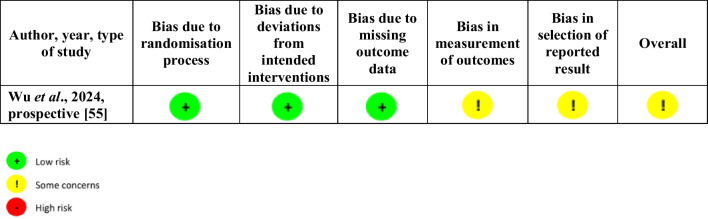
Assessment of risk of bias of the included articles based on Risk of Bias assessment-RoB-II [59]

## Discussion

The present study aims to report current evidence about using AI to identify anatomical structures during LC.

Even if the evaluation was heterogeneous among the included studies, AI models can be used to identify anatomical structures in the surgical field during LC, increasing surgical team awareness and playing a potential role in preventing BDI. [27, 32–55].

As reported and as expected, the AI ​​appears more effective in identifying areas of dissection, rather than individual structures (lower mAP value for CBD, CD, RS, and LEoLMLS, compared to Go and No-Go zone and CVS) [32, 37, 44]. Conversely, bACC values were also reported to be high for individual structures, reaching 3.8/4 (95%) in CBD recognition. Likewise, AI’s performance is reported grater for No-Go zone compared to Go zones [37, 38, 55].

These results can be explained because identifying dissection areas is certainly easier than a single anatomical structure. Equally, the volume of a No-Go zone within a frame is greater and therefore more easily identifiable compared to a Go zone [32–34, 27, 35–55].

It is reported in the literature that almost all BDIs are caused by errors in human visual perception and that technology is probably the most important weapon against these complications [61, 62]. AI models aim to create an accurate mental model that reflects the proper surgical anatomy, thanks to the segmentation of images collected from surgical videos [37–66].

While the agreement between AI and surgeons in identifying CVS reported in the included papers is high overall [33, 27, 35, 36, 50], it decreases significantly (to 56%) in cases of low-severity disease. Moreover, Korndorffer et al*.* reported considerably higher agreement (*p* < *0.001*) in more complex cases compared to simpler ones. This finding suggests that the nuances of the surgeon's decision-making process, especially in individual cases, may be difficult to capture within a single AI model [33].

The results reported in this systematic review demonstrate that the use of AI can lead to a shift in surgical strategy, resulting in safer dissections [43]. Furthermore, Leifman et al*.* show that intraoperative AI use results in an increased rate of achieving CVS (85% versus 19% in their standard LC and versus the 7–52% range reported in the literature) [41]. This result could be partially attributed to the well-known "Hawthorne effect", where the awareness of being monitored by AI potentially incentivized surgeons to optimize their performance [51].

It is crucial to differentiate between papers that use AI for post-hoc analysis of collected videos [33, 27, 35–37, 39, 41, 42, 45–48] and those that, departing from academic use, apply AI in clinical practice [32, 34, 38, 40, 43, 44, 49–55] and it is noteworthy that recent studies employ more complex technologies built upon extensive databases [49–55].

We strongly believe in the potential educational role of using AI during LC. As we know, and as reported in the literature the advice from a member of the surgical team other than the operator is one of the most adopted ways to prevent BDI and the risk of BDI appears lower in hospital with a surgical residency program and in high-volume centres, underlining the importance of the expert surgeon [67, 68]. We do not believe that AI can replace the opinion of an expert surgeon during a complex LC, but we assess that it could represent a valid tool especially for younger surgeons during MIS, since there is a lack of tactile sensitivity and visual information becomes predominant.

All the literature included in this review was published after 2020, highlighting the actuality and novelty of this paper, furthermore, although other systematic reviews on the role of AI in surgery exist in the literature [69–78], to our knowledge, this is the first to address its use in identifying anatomical structures during LC. This aspect represents an advantage and a limitation of this paper. Indeed, this is certainly a current topic, but there is active work in this field, with continuous publications, which has made it difficult to conduct a systematic review.

The main limitations of the present study are the overall poor quality and quantity of included studies and the presence of only one randomized control trials. The heterogeneity of the AI models employed, the heterogeneity of the inclusion criteria and the small number of cases analysed in each study represent further limitations. Expert opinion is also very heterogeneous, potentially limiting the generalizability of such models and the data was extracted from the articles themselves without access to the raw data, and this did not allow for true statistical analysis. Another important limitation is that the risk of BDI increases in some conditions such as high inflammation and fibrosis, bleeding, anatomical abnormalities, which also represent the exclusion criteria in many of the included papers [32, 34, 36, 38, 40, 43, 44, 48, 50, 54].

Finally, a further non-negligible limitation is that all the included studies coming from very few research groups with small number and possible overlapping dataset.

## Conclusions

Although current studies on using AI to identify anatomical structures during LC are few and with considerable heterogeneity, AI models seem to be valuable tools to anatomical identification, but we are still far from real-time guidance to minimize the risk of BDIs and others adverse events. The results seem promising, but new randomized trials and greater standardization are needed for these AI models to be reproducible.

## Supplementary Information

Below is the link to the electronic supplementary material.

Supplementary file1 (DOCX 13 KB)

Supplementary file2 (DOCX 27 KB)

## Data Availability

No datasets were generated or analysed during the current study.
