# Supplementary material for: Systematic review on the use of artificial intelligence to identify anatomical structures during laparoscopic cholecystectomy: a tool towards the future
Source: Langenbecks Arch Surg. 2025 Mar 18;410(1):101. doi: 10.1007/s00423-025-03651-6 (PMC11919950; doi:10.1007/s00423-025-03651-6)
Supplement: Supplementary file 1 — Supplementary file1 (DOCX 13 KB) [file 423_2025_3651_MOESM1_ESM.docx]

**Table 1. Keywords used for research in the PubMed, EMBASE, and Web of Science databases.**

| Artificial intelligence AND cholecystectomy |
| --- |
| Artificial intelligence AND cholecystectomy AND cholangiography |
| AI AND cholecystectomy |
| AI AND cholecystectomy AND cholangiography |
| CNN AND cholecystectomy |
| CNN AND cholecystectomy AND cholangiography |
| Convolutional neural network AND cholecystectomy |
| Convolutional neural network AND cholecystectomy AND cholangiography |
| Deep learning AND cholecystectomy |
| Deep learning AND cholecystectomy AND cholangiography |
| Machine learning AND cholecystectomy |
| Machine learning AND cholecystectomy AND cholangiography |
| Decision support systems AND cholecystectomy |
| Decision support systems AND cholecystectomy AND cholangiography |
| Biophysics-inspired algorithms AND cholecystectomy |
| Biophysics-inspired algorithms AND cholecystectomy AND cholangiography |
