# Supplementary material for: Systematic review on the use of artificial intelligence to identify anatomical structures during laparoscopic cholecystectomy: a tool towards the future
Source: Langenbecks Arch Surg. 2025 Mar 18;410(1):101. doi: 10.1007/s00423-025-03651-6 (PMC11919950; doi:10.1007/s00423-025-03651-6)
Supplement: Supplementary file 2 — Supplementary file2 (DOCX 27 KB) [file 423_2025_3651_MOESM2_ESM.docx]

**Table 2. Assessment of evidence of the included articles according to the Grading of Recommendations Assessment, Development and Evaluation (GRADE) [61].**

| **Certainty assessment** | | | | | | | **Impact** | **Certainty** | **Importance** |
| --- | --- | --- | --- | --- | --- | --- | --- | --- | --- |
| **№ of studies** | **Study design** | **Risk of bias** | **Inconsistency** | **Indirectness** | **Imprecision** | **Other considerations** |  |  |  |
| **Tokoyasu, 2020** | | | | | | | | | |
| 1 | non-randomised studies | serious | not serious | not serious | not serious | none | The intraoperative use of AI will help reduce the incidence of BDI, increasing the safety of LC | ⨁◯◯◯ Very low | IMPORTANT |
| **Korndorffer, 2020** | | | | | | | | | |
| 1 | non-randomised studies | serious | not serious | not serious | not serious | none | AI is a promising quality assurance tool. Disease severity has a significant impact on its use and surgeon oversight is still required to interpret the results. | ⨁◯◯◯ Very low | IMPORTANT |
| **Inomata, 2021** | | | | | | | | | |
| 1 | non-randomised studies | serious | serious | serious | serious | publication bias strongly suspected all plausible residual confounding would reduce the demonstrated effect | High DR of landmarks in case of mild to moderate inflammation of the gallbladder | ⨁◯◯◯ Very low | NOT IMPORTANT |
| **Mascagni, 2021** | | | | | | | | | |
| 1 | non-randomised studies | not serious | not serious | not serious | not serious | none | Deep learning models for workflow analysis can document CVS in LC with high accuracy | ⨁⨁◯◯ Low | IMPORTANT |
| **Mascagni, 2022** | | | | | | | | | |
| 1 | non-randomised studies | not serious | not serious | not serious | not serious | none | AI can identify CVS with a high degree of accuracy, although there is significant variability between different centres. | ⨁⨁◯◯ Low | IMPORTANT |
| **Mascagni, 2022** | | | | | | | | | |
| 1 | non-randomised studies | not serious | not serious | not serious | not serious | none | AI can automatically segment hepatocystic anatomy and assess the achievement of CVS in LC. | ⨁⨁◯◯ Low | IMPORTANT |
| **Madani, 2022** | | | | | | | | | |
| 1 | non-randomised studies | not serious | not serious | not serious | not serious | none | AI can be used to identify safe and dangerous zones of dissection, with high degree of performance. | ⨁⨁◯◯ Low | IMPORTANT |
| **Laplante, 2022** | | | | | | | | | |
| 1 | non-randomised studies | not serious | not serious | not serious | not serious | none | AI can be used to identify safe and dangerous zones of dissection during LC, with high specificity and PPV for Go zones, and high sensitivity and NPV for No-Go zones. | ⨁⨁◯◯ Low | IMPORTANT |
| **Colbeci, 2022** | | | | | | | | | |
| 1 | non-randomised studies | not serious | not serious | not serious | not serious | none | The MIL-AI model achieves 84% of accuracy in CVS assessment. | ⨁⨁◯◯ Low | IMPORTANT |
| **Nakanuma, 2022** | | | | | | | | | |
| 1 | non-randomised studies | not serious | not serious | not serious | not serious | none | The intraoperative use of AI in identifying anatomical landmarks during LC is useful | ⨁⨁◯◯ Low | IMPORTANT |
| **Ban, 2023** | | | | | | | | | |
| 1 | non-randomised studies | not serious | not serious | not serious | not serious | none | The AI model based on CGNN achieves 67% of average accuracy in CVS assessment | ⨁⨁◯◯ Low | IMPORTANT |
| **Endo, 2023** | | | | | | | | | |
| 1 | non-randomised studies | not serious | not serious | not serious | not serious | none | AI provide significant awareness to beginners and experts and prompted them to identify anatomical landmarks linked to reducing BDI. | ⨁⨁◯◯ Low | CRITICAL |
| **Fujinaga, 2023** | | | | | | | | | |
| 1 | non-randomised studies | not serious | not serious | not serious | not serious | none | AI provide landmark detection at appropriate situations, being effective in preventing BDI. | ⨁⨁◯◯ Low | IMPORTANT |
| **Kawamura, 2023** | | | | | | | | | |
| 1 | non-randomised studies | not serious | not serious | not serious | not serious | strong association | AI can evaluate CVS with high accuracy in real-time during LC, improving surgical safety. | ⨁⨁⨁◯ Moderate | IMPORTANT |
| **Alkhamaiseh, 2023** | | | | | | | | | |
| 1 | non-randomised studies | not serious | not serious | not serious | not serious | none | AI can potentially provide an intraoperative model for surgical video analysis and can support assessing the CVS during LC | ⨁⨁◯◯ Low | IMPORTANT |
| **Khalid, 2023** | | | | | | | | | |
| 1 | non-randomised studies | not serious | not serious | not serious | not serious | none | AI has potential to detect unsafe dissection and prevent BDIs through real-time intraoperative decision-support | ⨁⨁⨁⨁ High | CRITICAL |
| **Adrales, 2024** | | | | | | | | | |
| 1 | non-randomised studies | not serious | not serious | not serious | not serious | strong association | AI has good inter-rater reliability and has the potential to support efficient, objective assessments of surgical performance and competency. | ⨁⨁⨁◯ Moderate | IMPORTANT |
| **Hedge, 2023** | | | | | | | | | |
| 1 | non-randomised studies | not serious | not serious | not serious | not serious | all plausible residual confounding would reduce the demonstrated effect | The use of hierarchical task analysis for surgical video analysis could improve CVS assessment | ⨁⨁⨁◯ Moderate | IMPORTANT |
| **Smithmaitrie, 2024** | | | | | | | | | |
| 1 | non-randomised studies | not serious | not serious | not serious | not serious | strong association | AI effectively identifies anatomical landmarks in real-time during LC. | ⨁⨁⨁◯ Moderate | CRITICAL |
| **Petracchi, 2024** | | | | | | | | | |
| 1 | non-randomised studies | serious | not serious | not serious | not serious | strong association all plausible residual confounding would reduce the demonstrated effect | AI can detect the CVS in elective LC. | ⨁⨁⨁◯ Moderate | IMPORTANT |
| **Leifman, 2024** | | | | | | | | | |
| 1 | non-randomised studies | not serious | not serious | not serious | not serious | strong association | AI identifies CVS with very high accuracy in a real time OR setting, improving LC procedure’s safety and outcome. | ⨁⨁⨁◯ Moderate | CRITICAL |
| **Fried, 2024** | | | | | | | | | |
| 1 | non-randomised studies | not serious | not serious | not serious | not serious | strong association | Real- time use of AI facilitated a steady increase in CVS adoption and an improvement in OR efficiency. | ⨁⨁⨁◯ Moderate | CRITICAL |
| **Tashiro, 2024** | | | | | | | | | |
| 1 | non-randomised studies | not serious | serious | not serious | serious | publication bias strongly suspected | The combination of AI and ICG may reduce BDI rate | ⨁◯◯◯ Very low | IMPORTANT |
| **Wu, 2024** | | | | | | | | | |
| 1 | randomised trials | not serious | not serious | not serious | not serious | none | Significant improvement in CVS achievement in AI-enhanced feedback group as compared to self-learning group (11% to 78%, p=0.02). AI improved surgical performance and safety for novice surgeons during LC. | ⨁⨁⨁⨁ High | CRITICAL |
| **Protserov, 2024** | | | | | | | | | |
| 1 | non-randomised studies | not serious | not serious | not serious | not serious | none | AI-web platform able to predict safe and dangerous zones of dissection in real time during LC was built. | ⨁⨁◯◯ Low | IMPORTANT |

AI: artificial intelligence;BDI: bile duct injury; LC: laparoscopic cholecystectomy; DR: detection rate; CVS: critical view of Strasberg; PPV: positive predictive value; NPV: negative predictive value; MIL: : Multi Instance Learning; CGNN: Concept Graph Neural Networks OR: operating room; ICG: indocyanine green;
